# Implicit Motor Learning Strategies Benefit Dual-Task Performance in Patients with Stroke

**DOI:** 10.3390/medicina59091673

**Published:** 2023-09-16

**Authors:** Eito Arikawa, Masatomo Kubota, Tomoko Haraguchi, Masachika Takata, Shoji Natsugoe

**Affiliations:** 1 Graduate School of Health Sciences, Kagoshima University, 8-35-1, Sakuragaoka, Kagoshima 890-8544, Japan; e-arikawa@gyokushoukai.com; 2 General Rehabilitation Center, Kajikionsen Hospital, 4714, Kida, Kajiki, Aira City, Kagoshima 899-5241, Japan; 3 Department of Occupational Therapy, School of Health Science, Factory of Medicine, Kagoshima University, 8-35-1, Sakuragaoka, Kagoshima 890-8544, Japan

**Keywords:** stroke, implicit learning, explicit learning, serial reaction time, attention, working memory, dual-task, motor skills, rehabilitation

## Abstract

*Background and Objectives*: In stroke rehabilitation, the use of either implicit or explicit learning as a motor learning approach during dual tasks is common, but it is unclear which strategy is more beneficial. This study aims to determine the benefits of implicit versus explicit motor learning approaches in patients with stroke. *Materials and Methods*: Seventeen patients with stroke and 21 control participants were included. Motor learning was evaluated using the Serial Reaction Time Task (SRTT) in the context of dual-task conditions. The SRTT was conducted on two separate days: one day for implicit learning conditions and the other day for explicit learning conditions. Under the explicit learning conditions, a task rule was given to the participants before they started the task, but not under the implicit learning conditions. Learning scores were calculated for both implicit and explicit learning, and these scores were then compared within groups for patients with stroke and controls. We calculated the difference in learning scores between implicit and explicit learning and conducted a correlation analysis with the Trail Making Test (TMT) Parts A and B. *Results*: Learning scores on the SRTT were not different between implicit and explicit learning in controls but were significantly greater in patients with stroke for implicit learning than for explicit learning. The difference in learning scores between implicit and explicit learning in patients with stroke was correlated with TMT-A and showed a correlation trend with TMT-B. *Conclusions*: Implicit learning approaches may be effective in the acquisition of motor skills with dual-task demands in post-stroke patients with deficits in attention and working memory.

## 1. Introduction

Improving dual-task performance is important in stroke rehabilitation [1,2]. This is because many activities of daily life are performed in dual-task situations [3]. For example, talking while walking and enjoying music while operating a smartphone are both based on a combination of two cognitive skills [2,3]. These activities are realized by unconsciously performing a series of movements [4,5,6]. In other words, when a series of actions are automated, we can carry on with our daily lives without being aware of what is being done. However, in patients with stroke, daily life may be disrupted due to severe impairment of dual-task performance [7,8]. Although this may not be the case for all patients with stroke, impaired attention and working memory are more likely to result in decreased ability for dual-task skills and reduced performance [9].

Implicit learning approaches have shown promise in improving dual-task performance in patients with stroke [10,11]. Implicit learning refers to learning that occurs in the absence of awareness and declarative knowledge of the motor task performed [12] and is defined as the process of unconscious learning [13]. Previous studies suggest that patients with stroke can implicitly learn on the non-paralyzed upper extremity [10,14,15]. Also, the performance of implicitly learned tasks appears to be robust against the interference of secondary tasks under dual-tasking [16,17].

Furthermore, implicit learning is expected to minimize the load on working memory because it does not require conscious processing [10,11]. For example, in stroke rehabilitation, when motor skills are acquired by explicitly controlling the patient’s movements, the early stages of motor learning require working memory resources [10,12,18]. At this stage, declarative knowledge of motor skills is acquired in order to consciously control motor performance [10]. This is called explicit learning, as opposed to implicit learning [12,18]. When movement patterns are automated through movement repetition, learners can automatically perform a series of movements without being conscious of it [19,20], and motor skills are fine-tuned; that is, they are acquired as procedural knowledge [10] and do not require conscious control and therefore do not need to rely on working memory [10].

Given that implicit learning does not depend on working memory, this could be a promising strategy for stroke rehabilitation [10]. For example, in patients with stroke, motor tasks such as walking may require conscious motor monitoring and control [10]. In the case of post-stroke patients with deficits in attention and working memory, the strategy of conscious control of movement may lead to inefficient learning due to the severe demands on working memory.

Although many studies of implicit learning in patients with stroke have been conducted, studies demonstrating that implicit learning is useful in stroke rehabilitation are still lacking [10,21]. Patients with stroke who have deficits in attention and working memory may also develop dual-task deficits [9], but it is not clear whether implicit or explicit learning can improve dual-task performance. Since the two types of motor learning involve different processing pathways and brain resources, it is necessary to distinguish between them before using them in stroke rehabilitation [12]. Thus, verifying whether implicit or explicit learning is effective under dual-task conditions is highly likely to enhance stroke rehabilitation.

The serial reaction time task (SRTT), developed by Nissen and Bullmer, is the standard method for examining implicit learning [12,20,22]. To date, it has been the most applied method in the study of implicit learning in patients with stroke [10,18]. The purpose of this study was to reveal the difference in learning effects between implicit and explicit learning under dual-task conditions in patients with stroke. We expected the effect of implicit learning to be greater than that of explicit learning under dual-tasking.

## 2. Materials and Methods

### 2.1. Participants

We enrolled 17 patients who had experienced a stroke (mean age SD standard deviation: 63.4 SD ± 9.4 years) and 21 healthy controls, by reference to the previous studies [15,17]. In the stroke group, an average of 31.1 months (SD 49.4) had elapsed since onset. Of the 17 patients in the stroke group, 12 had right hemisphere injuries and five had left hemisphere injuries. Patients with stroke were recruited at the Kajiki Onsen Hospital and related facilities. We assured all participants that their participation was voluntary and completely anonymous. Patients needed to have been diagnosed with a hemisphere injury, absence of aphasia, absence of visual or hearing problems, and mild stroke severity (National Institutes of Health Stroke Scale score < 5). We excluded patients with unilateral spatial neglect that interfered with activities of daily living or those with cognitive ailments (Mini-Mental State Examination score < 24). Patients with any missing data were also excluded. Healthy volunteers aged from 50 to 71 years were recruited as the control group; their inclusion criteria were no history of neurological or psychiatric disorders.

### 2.2. Characteristics of Attention and Working Memory

The Trail Making Test Part A (TMT-A), the TMT Part B (TMT-B), and the Paced Auditory Serial Addition Task (PASAT) were used to evaluate participant attention. The TMT is the most commonly used neuropsychological test and reflects processing speed, visuospatial exploration, flexible attention switching, and working memory [23,24]. PASAT is one of the most frequently used tests for evaluating attention processing and is used to measure divided attention and working memory [25,26,27,28]. MMSE is a widely used cognitive screening test for patients with stroke with the ability to assess memory, orientation, attention, registration, language, and visual construction [29]. A recommended cut-off score is <24 [29].

### 2.3. Procedures and Tasks

**Figure 1:**
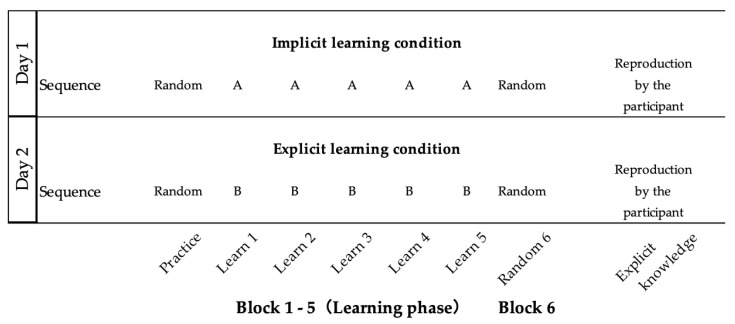
Experimental protocol of implicit learning and explicit learning using the serial reaction time task. Each learning protocol consisted of a practice block, a learning phase (Blocks 1–5), and a random block (Block 6). Sequence A, 1–3–4–2–3–2, and sequence B, 3–1–2–4–1–4, were used in implicit and explicit learning, respectively.

Differences in learning effects between implicit and explicit learning were compared using SRTT under dual-task conditions in this study. In Figure 1, the configuration of the experiment is explained to the readers using diagrams, illustrating each stage. These stages were as follows: (1) one practice block; (2) five learning blocks (e.g., sequence A: 1-3-4-2-3-2); (3) one random block (Block 6); and (4) the stage of re-enacting the learned sequences. Under the explicit learning conditions, a task rule was given to the participants before they started the task, but not under the implicit learning conditions. All participants received SRTT under two conditions: implicit learning (Day 1) and explicit learning (Day 2), on two separate days. In order to perform the SRTT under implicit learning conditions without any knowledge of the rule, the implicit learning condition was carried out first. Furthermore, with each condition, SRTT was held at intervals of 48 h or more to eliminate any influence of an order effect [30].

The SRTT was created using Microsoft Excel 2019 Visual Basic for Applications (Microsoft Corp, Redmond, WA, USA). Four squares were displayed horizontally on a personal computer monitor, and an asterisk was displayed on one square. Participants were asked to press, as quickly as possible, one of the four buttons on a SuperLab software (Cedrus, San Pedro, CA, USA) corresponding to the asterisk’s position using their index finger on the unaffected side. When participants pressed the wrong button, a beep sounded. The asterisk continued to be displayed until the correct button was pressed.

The order of the sequence was “1-3-4-2-3-2 (sequence A)” for the implicit learning condition and “3-1-2-4-1-4 (sequence B)” for the explicit learning condition, based on the report of Schmitz et al. [31]. “One” corresponded to the leftmost square, and “four” corresponded to the rightmost square. The stimulation interval was 500 ms. The learning phase consisted of 5 blocks. In each block, the 6-item series was repeated 10 times, as previously described [32].

A tone-counting task was used as the secondary task [33,34]. A tone-counting task is sufficient to elicit a dual task [19]. During the dual-task SRTT, we instructed the participants that a pure tone of 1000 Hz would be randomly played 20–40 times per session. The pure tone lasted 0.5 s. We asked the participants to report how many times the sound was played after the end of each block. The tone counting task was executed in all blocks, including the practice and 6th blocks.

### 2.4. Explicit Knowledge Confirmation

Under the implicit learning condition, after completion of the random block (6th block), we asked the participants about any observations they made during the task to confirm their awareness of the existence of sequence rules. In addition, to identify the effect of explicit knowledge, we requested a re-enactment of the repeated sequence shown after Block 6. In the re-enactment, the first number of the sequence (e.g., “1” for sequence A) was initially displayed on the screen, followed by a subsequent series of sequences. For this session, we instructed participants to focus on the accuracy of sequence re-enactment rather than the response speed. Re-enactment was required under both conditions.

### 2.5. Data Analysis

Pressing the wrong answer button was defined as an incorrect attempt, and the error ratio was calculated to verify the accuracy of the response. The error ratio was calculated by dividing the number of incorrect answer selections by the total count of responses and was defined as a percentage from Block 1 to Block 6. The time from the appearance of the asterisk to the pressing of the correct answer button was calculated as reaction time (RT), and we calculated the median RT for each block. We also calculated the mean value across all blocks.

The learning score was defined as the change in reaction time between the 5th series block and the following random block (6th block). Implicit learning in the SRTT paradigm is indicated by a significant extension of the reaction time of the last random block in a series of trials compared to the previous block. This occurs because the absence of implicitly learned sequences leads to poor task performance [18,22]. The learning score was calculated by subtracting the median Block 5 RT from the median Block 6 RT. A large learning score indicated a large learning effect. In the re-enactment, the average value was calculated.

We performed statistical analysis using the statistical software package SPSS 26 (IBM, Armonk, NY, USA). The normality of the distribution of each data set was tested using the Shapiro–Wilk test, and we used the appropriate parametric or nonparametric tests to determine statistical significance. A *p*-value of <0.05 was considered statistically significant. We investigated the differences in performance between the stroke group and the control group using the independent sample *t*-test or the Mann–Whitney U test.

For the analysis, we used a repeated measures two-way ANOVA that included blocks (Block 5 vs. Block 6) and learning types (implicit learning vs. explicit learning) as variables of the intra-participant factors. When an interaction was observed, the learning score, depending on the learning type, was compared using the paired-samples *t*-test or the Mann–Whitney U test in each group. In the re-enactment, we analyzed the number of reproduced items using the independent sample *t*-test or the Mann–Whitney U test.

To confirm the effect of attention deficits on the learning effect depending on learning type, the correlations among the differences between implicit and explicit learning scores (TMT-A, TMT-B, and PASAT) were analyzed using Spearman’s correlation or Pearson’s correlation with two-tailed tests.

The effect size was set according to Cohen’s effect size as follows: small for d or r = 0.10; medium for d or r = 0.30; and large for d or r = 0.50; small for η^2^ = 0.01; medium for η^2^ = 0.06; and large for η^2^ = 0.14. All results are expressed as the mean ± standard error.

## 3. Results

### 3.1. Participants

Table 1 shows the demographic characteristics of patients and the results of the Mini-Mental State Examination (MMSE), TMT-A, TMT-B, and PASAT. TMT-A and TMT-B could not be tested in 1 of 17 patients with stroke. Significant differences were observed between the patients with stroke and control groups in TMT-A (U = 270.50, *p* < 0.01), TMT-B (U = 297.50, *p* < 0.01), and PASAT (t = 2.20, *p* = 0.033). MMSE was not significant between patients with stroke and the control group (U = 117.00, *p* = 0.065). All except one patient with a left hemisphere injury were right-handed. Of the five patients with left hemisphere injury, two had right hemiplegia. Table 2 shows the causative disease, the main lesion, the presence or absence of hemiplegia, and the number of months since onset. The 21 healthy controls were age 66.0 ± 5.7 years. There was no significant age difference between patients with stroke and healthy controls (U = 209.50, *p* = 0.367).

### 3.2. Error Rates and Total Mean Reaction Times

Table 3 shows the average RT of all blocks in the two groups. Error rates in both groups were generally low, at <2% in the stroke group and <1% in the control group. As shown in Table 3, the stroke group performed longer than the control group under all conditions, and the difference was significant (implicit learning: t = 5.72, *p* < 0.001, d = 1.87; explicit learning: U = 313.00, *p* < 0.001, r = 0.64).

### 3.3. Reaction Time and Learning Score

**Figure 2:**
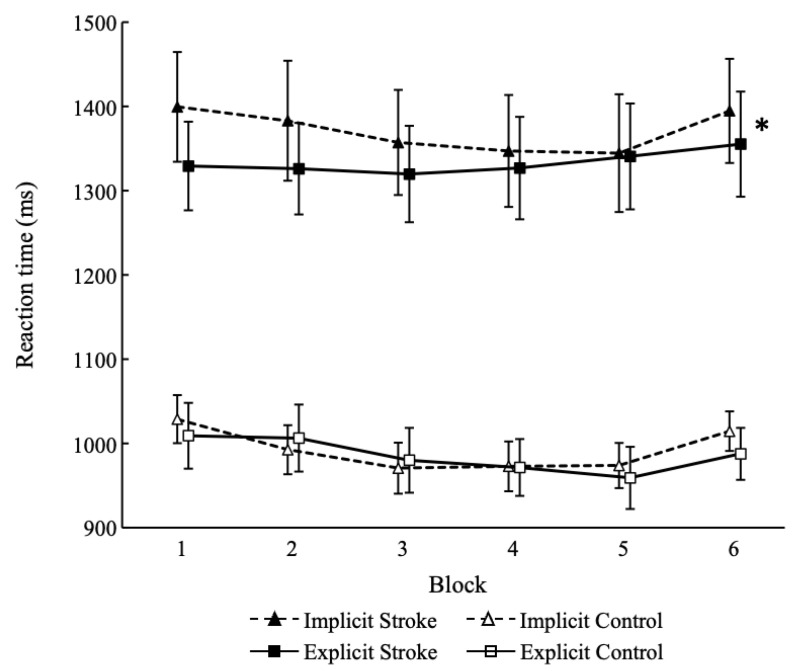
Results for the serial reaction time task. * indicates a significant difference between implicit learning and explicit learning (*p* < 0.05). Stroke, patients with stroke; Control, healthy participants.

The results for the serial reaction time task is presented in Figure 2. The reaction time interactions were significant between implicit learning and explicit learning in the stroke group (F [1, 16] = 8.257, *p* = 0.011, η^2^ = 0.04). The difference between the learning scores of the dual-task implicit learning condition and the explicit learning condition in the stroke group was significant (t = 2.87, *p* = 0.011, d = 0.58). However, in the control group, the learning score interactions were not significant between the two (F [1, 20] = 0.79, *p* = 0.384, η^2^ = 0.00). A significant effect was observed in the block (F [1, 20] = 24.07, *p* < 0.001, η^2^ = 0.017), but not in the learning type (F [1, 20] = 0.95, *p* = 0.340, η^2^ = 0.006).

### 3.4. Correlation

The differences in learning scores between implicit and explicit learning were correlated with TMT-A in the stroke group, while TMT-B showed a correlation trend (TMT-A: rs = −0.514, *p* = 0.042; TMT-B: rs = −0.451, *p* = 0.079). PASAT showed no correlation (PASAT: rs = −0.057, *p* = 0.826). In the control group, TMT-A, TMT-B, and PASAT all showed no correlation (TMT-A: rs = −0.268, *p* = 0.239; TMT-B: rs = −0.105, *p* = 0.650, PASAT: rs = 0.206, *p* = 0.369).

### 3.5. Explicit Knowledge

When investigating the influence of explicit knowledge on learning, we observed non-significant differences between the two groups for implicit learning (stroke group: 1.9 ± 0.3, control group: 2.7 ± 0.2; U = 126.00, *p* = 0.128, r = −0.26) and explicit learning (stroke group: 2.3 ± 0.3, control group: 2.9 ± 0.3; t = 1.21, *p* = 0.117, d = 0.39).

## 4. Discussion

In this study, we compared implicit learning and explicit learning under dual-task conditions in patients with stroke and controls and investigated the difference in learning effects. Our results showed that in the control group, there was no difference in learning scores between implicit and explicit learning, and there was no interaction, whereas in the stroke group, implicit learning had a higher learning score than explicit learning, indicating an interaction. Therefore, it is suggested that patients with stroke may benefit from strategies of implicit learning when acquiring motor skills in dual-task situations.

Implicit motor learning may therefore be beneficial in stroke rehabilitation and may be particularly beneficial for post-stroke patients with deficits in attention and working memory. First, several previous studies examining motor learning in stroke patients have shown that implicit learning is superior to explicit learning [10]. For example, Boyd et al. [35] report that explicit presentation of motor sequence information interferes with implicit motor learning in post-stroke patients and may impede performance. Orrell et al. [17] also suggest that using verbal components of working memory to control motor activity may interfere with motor acquisition in patients with stroke. Interestingly, the performance of motor skills acquired by implicit learning was stable and was maintained long-term even under dual-task conditions [17]. Second, all participants in these previous studies had high MMSE scores, with no difference between patients with stroke and controls [14,15,17,35,36,37,38]. In the present study, participants’ MMSE scores did not differ between patients with stroke and controls, as in previous studies. On the other hand, scores on tests measuring attention and working memory, such as TMT-A, TMT-B, and PASAT, were lower in patients with stroke than in controls. Previous studies have not mentioned such measures of attention and working memory [14,15,17,35,36,37,38]. The results of this study showed that the differences between implicit and explicit learning scores were correlated with TMT-A scores; furthermore, TMT-B scores also showed a correlation trend. This suggests that implicit learning may be more beneficial for motor learning than explicit learning in patients with stroke who have deficits in attention and working memory.

The majority of implicit learning studies using SRT tasks show that implicit learning and working memory are independent [18]. For example, Unsworth and Engle [39] measured working memory capacity with an operation span task and found no difference in the performance of implicit learning between those with high individual variability and those with low individual variability. Bo et al. [40] and Bo et al. [41] also report no correlation between SRT task learning scores and working memory measures. In other words, implicit learning can be interpreted as being less affected by attention and working memory.

One caution might be required in interpreting these results. The dual-task condition itself may implicitly enhance learning [10,11,12]. In the years since Masters [42] first mentioned the dual-task paradigm as an implicit learning strategy, there have been studies along this line [10,11]. As a result of the current study, both groups learned sequences under both implicit and explicit learning conditions, but there was no difference in explicit knowledge between the two conditions in either group. Therefore, even if the dual-task situation was explicit (when there was knowledge of the rules), an element of implicit learning may have been involved; that possibility cannot be denied. However, more importantly, the results of the current study showed that the stroke group had higher learning scores in the implicit learning condition than in the explicit learning condition. The significant difference between the two conditions was whether or not there was awareness of the sequences. It is widely accepted that implicit and explicit learning are distinguished as unconscious and conscious learning, respectively [12]. In other words, patients with stroke are more likely to have stable motor performance if they are not conscious of the order of the motor sequence when acquiring motor skills in a dual-task situation.

### Strengths and Limitations

The clinical utility of this study is the value of incorporating implicit learning strategies into programs during stroke rehabilitation when attention and working memory deficits are suspected. Patients with stroke are more likely to have conscious control over their movements [10,19], and those with more conscious control have better cognitive resources such as attention and working memory [19]. In particular, patients with suspected attention or working memory deficits are less likely to succeed in dividing and allocating their resources [43,44,45]. Implicit learning strategies may therefore be useful in enhancing the learning of motor sequences in patients with stroke who rely on attention and working memory to control their movements. In addition, it is necessary to consider that we encounter various dual-task situations in our daily lives. Skills acquired through implicit learning are thought to be executed involuntarily, but it is important that they are executed in every dual-task situation in daily life. Implicit learning strategies are suggested to be useful in assisting patients with impaired attention and working memory to acquire motor skills during dual-task training in stroke rehabilitation.

Furthermore, to effectively utilize implicit learning strategies, it is imperative to comprehend the neural networks implicated in motor learning and consider the brain regions affected by stroke. Essential components of the neural network implicated in motor learning comprise the primary motor cortex, premotor cortex, supplementary motor cortex, basal ganglia, prefrontal cortex, and posterior parietal cortex (PPC) [12]. The dorsolateral prefrontal cortex (DLPFC) and the PPC play a critical role in the early stages of motor learning, and these areas form a corticostriatal loop with strong connections to the anterior part of the basal ganglia. The early learning phase involves explicit learning, which requires active attention and working memory [18]. Therefore, it may be advisable to employ an implicit learning strategy in the treatment of stroke patients with injuries to the corticostriatal loops, involving the DLPFC and PPC.

In addition, an early learning phase transitions to a second phase in which motor sequences are stabilized. This phase is called the “integration phase”, and is mainly accomplished by the basal ganglia [12]. It is reported that explicit information hinders implicit motor learning in patients with basal ganglia injuries [37], yet it is unclear whether utilizing available attentional resources for explicit information is disadvantageous for motor learning [12]. If the DLPFC and PPC are intact, the available attentional and working memory resources may be used for explicit information during motor learning. In this situation, it may be necessary to consider the use of implicit learning strategies.

The current research has some limitations. First, the characteristics of the participants were non-uniform. In particular, the site of brain injury was diverse, and some patients had damage to the cerebral cortex, subcortex, or both. In motor sequence learning studies, information on inter-study lesion heterogeneity and lesion location is unclear in many studies [12], and this needs to be clarified. In addition, the number of months elapsed from stroke onset ranged from 1 to 191 months, from the acute stage to the chronic stage. Therefore, given that the stage of stroke recovery may be a determinant of patient performance, it is possible that the participants’ number of months since stroke onset influenced the results. Furthermore, regarding the order of sessions, implicit learning was prioritized over explicit learning for all participants. It is usually desirable to start with a counterbalance. However, if this order were reversed (explicit learning takes precedence over implicit learning), it could lead to bias in predicting the existence of rules in conditions of explicit learning. Finally, the small sample size of this study might make it difficult to generalize the present results. Studies involving larger samples of dual-task implicit learning in patients with stroke are warranted for confirmation and extension of the current evidence.

## 5. Conclusions

In this study, the effects of implicit motor learning approaches on the performance of motor learning under dual-task conditions in patients with stroke were investigated. Results showed that patients with stroke demonstrated better motor learning under implicit than explicit motor learning conditions compared to controls. Patients with stroke also had inferior attention and working memory abilities compared to controls. Therefore, it is suggested that rehabilitation of patients with stroke with impaired attention and working memory may benefit from an implicit motor learning approach when performing dual-task training.

## Figures and Tables

**Table 1:** Demographic characteristics of patients and the results of the Mini-Mental State Examination (MMSE) and Paced Auditory Serial Addition Task (PASAT).

	Patients with Stroke (*n* = 17)	Healthy Controls (*n* = 21)	*p*-Value
Mean age (years)	63.4 (±9.4)	66.0 (±5.7)	0.360
Male/female	8/9	6/15	
TMT-A (s) **	71.6 (±29.7)	40.1 (±13.2)	0.001
TMT-B (s) **	126.6 (±57.0)	63.5 (±15.8)	0.001
PASAT (%) *	45.2 (±20.6)	59.0 (± 17.2)	0.033
MMSE	27.2 (±1.9)	28.3 (±1.7)	0.065
NIHSS	2.6 (±1.8)	NA	

Means and standard deviations (in parenthesis) are provided. TMT-A, Trail Making Test Part A; TMT-B, Trail Making Test Part B; PASAT, Paced Auditory Serial Addition Test; MMSE, Mini-Mental States of Examination; NIHSS, National Institutes of Health Stroke Scale; NA, not applicable. * indicates *p* < 0.05; ** indicates *p* < 0.01.

**Table 2:** Lesion location for 17 participating patients with stroke.

Participant	Lesion Side	Etiology	Lesion Location	Hemiparesis	Time Since Onset (Months)
1	Right	Ischemic	Temporal/Parietal		14
2	Left	Hemorrhagic	Frontal	None	23
3	Right	Hemorrhagic	Frontal/Parietal	None	1
4	Right	Hemorrhagic	Putamen		191
5	Right	Hemorrhagic	Parietal/Putamen		75
6	Right	Hemorrhagic	Frontal/Putamen		68
7	Right	Ischemic	Putamen		84
8	Right	Ischemic	Frontal/Temporal/Parietal		21
9	Right	Hemorrhagic	Thalamus		6
10	Right	Hemorrhagic	Frontal	None	4
11	Right	Hemorrhagic	Temporal		11
12	Left	Ischemic	Putamen		3
13	Left	Ischemic	Putamen		3
14	Left	Ischemic	Globus pallidus	None	16
15	Left	Hemorrhagic	Thalamus	None	2
16	Right	Hemorrhagic	Putamen		2
17	Right	Ischemic	Putamen		4

**Table 3:** Motor sequence learning: relevant parameters of the SRT task.

Group	Condition	Total Mean RT (ms)	Error Rate (%)	Learning Score (RT Block 6–RT Block 5) (ms)	Explicit Knowledge (No. of Items)
Stroke	Implicit	1366.2 ± 64.9 **	1.0 ± 0.1	50.1 ± 12.7	1.9 ± 0.3
	Explicit	1328.0 ± 56.9 **	0.6 ± 0.1	14.6 ± 11.8	2.3 ± 0.3
Control	Implicit	987.6 ± 28.0	0.0 ± 0.0	40.6 ± 10.0	2.7 ± 0.2
	Explicit	985.1 ± 37.1	0.0 ± 0.0	28.6 ± 9.5	2.9 ± 0.3

Mean and standard error of the mean are presented separately for post-stroke patients and controls. Total mean RT shows the average value of blocks 1 to 5. Stroke, post-stroke patients; Control, healthy participants; RT, reaction time. ** indicates *p* < 0.01, vs. control.

## Data Availability

The data presented in this study are available on request from the corresponding author.
